# Fabrication of Durably Superhydrophobic Cotton Fabrics by Atmospheric Pressure Plasma Treatment with a Siloxane Precursor

**DOI:** 10.3390/polym10040460

**Published:** 2018-04-22

**Authors:** Jing Yang, Yi Pu, Dagang Miao, Xin Ning

**Affiliations:** Industrial Research Institute of Nonwovens and Technical Textiles, College of Textiles and Clothing, Qingdao University, Qingdao 266000, China; 15764238363@163.com (J.Y.); puyiconan@163.com (Y.P.)

**Keywords:** atmospheric pressure plasma, durability, superhydrophobility, cotton fabric, siloxane

## Abstract

The surface treatment of fabrics in an atmospheric environment may pave the way for commercially viable plasma modifications of fibrous matters. In this paper, we demonstrate a durably superhydrophobic cotton cellulose fabric prepared in a single-step graft polymerization of hexamethyldisiloxane (HMDSO) by N_2_ and O_2_ atmospheric pressure plasma. We systematically investigated effects on contact angle (CA) and surface morphology of the cotton fabric under three operational parameters: precursor value; ionization gas flow rate; and plasma cycle time. Surface morphology, element composition, chemical structure and hydrophobic properties of the treated fabric were characterized by scanning electron microscope (SEM), EDS, FTIR and CA on the fabrics. The results indicated that a layer of thin film and nano-particles were evenly deposited on the cotton fibers, and graft polymerization occurred between cellulose and HMDSO. The fabric treated by O_2_ plasma exhibited a higher CA of 162° than that treated by N_2_ plasma which was about 149°. Furthermore, the CA of treated fabrics decreased only 0°~10° after storing at the ambient conditions for four months, and treated fabrics could also endure the standard textile laundering procedure in AATCC 61-2006 with minimum change. Therefore, this single-step plasma treatment method is shown to be a novel and environment-friendly way to make durable and superhydrophobic cotton fabrics.

## 1. Introduction 

Surface superhydrophobicity has always attracted tremendous attention due to its potential applications in multiple fields including self-cleaning [1,2] and oil-water separation [3,4,5]. Water droplets on the superhydrophobic surface could maintain almost spherical shape and easily roll off to remove the dirt particles in their path [6], which can make the fabric self-cleaning and save a large amount of water needed for washing in daily life. Many methods have been reported to fabricate superhydrophobic fabrics: gas phase coating procedure [6,7]; layer-by-layer assembly [8]; electrospinning [9]; plasma polymerization [10,11,12]; and others. Among them, plasma polymerization offers many advantages over traditional treatments, including that it is water-free, a clean process, cost effective and there is no discharge of waste solutions. Plasma induced surface modifications (including polymerization) do not damage the mechanical properties of the fiber and bulk fabrics, because the depth of modification on the fiber surface is just less than ~10 nm [13]. Furthermore, recent technological advances in atmospheric pressure plasma generation and applications have tremendously improved the commercial viability of the traditional vacuum plasma technology in allowing continuous operation at a regular plant environment while preserving many of the benefit of plasma particle/substrate interactions with the surface being modified [14].

David J. Marchand [11] has reported that superhydrophobicity on silicon wafers and glass slides was achieved by a single-step process of atmosphere plasma with a tetramethylsilane (TMS) precursor. Ahmed El-Shafei [15] has prepared durable and hydrophobic cotton fabric via atmospheric pressure plasma polymerization of a vapor deposited fluorocarbon mixture, and has also studied the influence of monomer flow rate and plasma preactivation. Chaio-Ru Hsiao [16] investigated plasma-polymerized superhydrophobic coating material formed by controlling the hexamethyldisiloxane (HMDSO)/CF4 monomer flow ratio at low pressure condition. Esmeryan [17] has researched modification of the carbon soot through polymerization of HMDSO by means of glow discharge RF plasma. Shuai Liu [18] has prepared durable superhydrophobic fabric through post-crosslinking of silane achieved by vacuum plasma in Argon. Ricardo Molina [19] has studied the preparation of hydrophobic coating using plasma technology in a liquid fluorinated monomer. Pengyun Xu and Larry Pershin [20] have fabricated superhydrophobic ceramic coatings by a one-step solution precursor plasma spray process.

However, there is little prior literature on fabricating durable and superhydrophobic fabrics by atmospheric pressure plasma in a one-step process. In addition, we have not seen a systematic investigation on the influences of various process parameters on efficacy of surface morphology, and the water contact angles on said substrate. It is from this understanding that we have undertaken the present study to explore the technical benefit of atmospheric treatment on cotton fabrics and its commercial feasibility. The cotton substrate was chosen because its ubiquitous presence in our daily life and the widely scientific implications of studying the plasma interaction with the cellulosic structures. And the objective of a durably superhydrophobic cotton fabric is directed towards the eventual application of this technology in the real world. 

In the following sections, we will demonstrate a simple-step scheme to fabricate the superhydrophobic, stable as well as durable coating on cotton fabric via plasma polymerization of HMDSO at atmospheric pressure. The role of N_2_ and O_2_ ionization gas, plasma cycle time, precursor value and ionization gas flow rate on the contact angle (CA) and surface morphology of the treated fabric will be systematically investigated. The durability and stability of the superhydrophobic coating were measured using the standard AATCC methods. In addition, element content, chemical structure and hydrophobic properties of the treated fabric were characterized by scanning electron microscope (SEM), EDS and FTIR.

## 2. Experimental

### 2.1. Materials

HMDSO as a graft precursor was purchased from Aladdin (Shanghai, China) (99%) and used as received. Commercial cotton fabrics (plain weave, 145 g/m^2^) were obtained from Weifang Xinhui. All the fabrics were rinsed with acetone (99.9%) and cleaned by ultrasonic for 15 min, subsequently washed with deionized water for three times and dried in oven at 60 °C for 30 min before deposition.

### 2.2. Plasma Polymerization

**Figure 1:**
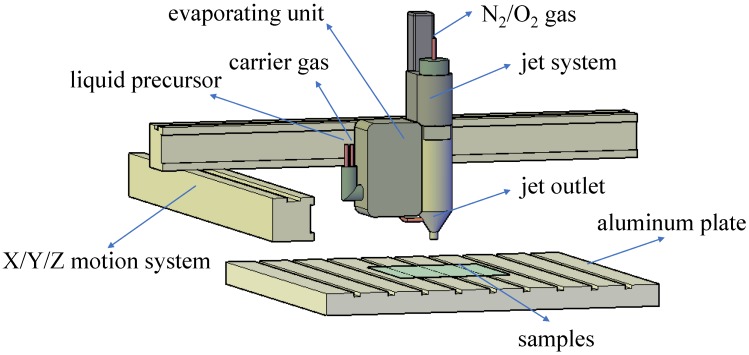
A schematic diagram of the atmospheric pressure glow discharge plasma system.

Plasma polymerization was carried out using the Atmospheric Pressure Plasma System Model AS400+PFW10 manufactured by Plasma Treat GmbH (Steinhagen, Germany). A schematic diagram of the system is shown in Figure 1, it is a glow-discharge plasma work space having dimensions of 33 cm by 12.5 cm, and the plasma system is powered by an RF power (19 kHz) supply and matched network.

Nitrogen (N_2_, 99.7% purity) and oxygen (O_2_, 99.7% purity) were used to generate plasma, the HMDSO liquid as a precursor passed through the evaporating unit and mixed with a precisely metered carrier gas in a steam state and fed to the jet outlet. Argon gas carried the precursor vapors to the jet outlet at a flow rate of 300 L/h. The most precise mass flow rates are ensured with the aid of the integrated mass flow meter acting in conjunction with the control system. The substrates were fixed on an aluminum plate and the plasma jet was controlled by a X/Y/Z motion system. In the N_2_ or O_2_ plasma, the active atoms and molecules can dissociate the HMDSO and easily interact with the organic moieties of monomer fragmentation, which can finally generate a hydrophobic coating. The plasma voltage, jet moving speed and raster offset were 280 V, 5 m/min and 2 mm, respectively, the distance between substrate and jet nozzle was kept constant at 4 cm.

The details of three operational parameters (precursor value, ionization gas flow rate, plasma cycle time) are shown in the Table 1. All of the experiments were carried out in the ambient conditions.

### 2.3. Characterization

Contact angle and slide angle were measured on a contact angle goniometer (JY-PHb). A drop of distilled water of 10 μL volume was placed on the fabric sample, and the contact angle values were carried out after the water drops was kept on the surface of the treated samples for 60 s. All the CA and SA values were reported as the mean of three measurements.

The wetting time of a water droplet on the surface of the treated fabric was measured, an ionized water droplet of 10 μL was allowed to fall from a 5 cm height onto the cotton fabric surface, the averages of wet-out times at five different places on the sample surface were recorded.

In order to determine the air permeability of the plasma treated cotton fabric, experiments were conducted at an air pressure of 200 Pa through the fabric area of 20 cm^2^ on a FX3300 Lab air permeability tester. An average of 10 readings was recorded.

Surface morphology of the treated fabrics was observed using a Phenom Pro Scanning Electron Microscope (SEM) (JEOL Ltd., Tokyo, Japan) operated at an acceleration voltage of 10.0 kV, after an Au sputtering for 60 s. Images were recorded at 5000 and 10,000 times magnification. For all the samples the experiments were repeated for three times and the SEM images presented in the manuscript are representative for all repeated experiments.

Fourier Transform Infrared spectroscopy measurements were performed on a Nicolet iS5 (Waltham, MA, USA) bench unit with OMNIC software Version 7.2 (Waltham, MA, USA) using an OMNI-ATR-Sampler with a diamond crystal. Spectra were obtained with an average of 32 scans using a resolution of 4 cm^−1^.

The SEM-EDS analyses were carried out using an JSM-7800F Scanning Electron Microscope (JEOL Ltd., Tokyo, Japan) coupled to a X-Max Energy-Dispersive X-ray spectrometer (JEOL Ltd., Tokyo, Japan) for electron image acquisitions and elemental analysis (punctual and imaging) respectively.

### 2.4. Durability

The durability of the treated fabrics was evaluated by washing the fabrics using a method specified to AATCC (American Association of Textile Chemists and Colorists) Test Method 61-2006 Test No. 1A. During washing, the samples were washed by using a standard Launder-ometer (SW-20B, Quanzhou Meibang Instrument CO. Ltd., Quanzhou, China) equipped with stainless-steel lever-lock canisters (75 × 125 mm). The fabric sample (size, 50 × 100 mm) was placed in a 200 mL aqueous solution containing 0.37% (*w*/*w*) AATCC standard reference detergent without optical brightener and 10 stainless steel balls. During laundering, the temperature was controlled at 42 °C, and the stirring speed at 40 ± 2 rpm. After 45 min of laundering, the laundered sample was rinsed with deionized water, and then dried at 60 °C for 50 min without spinning. The contact angle and sliding angle were then measured. This standard washing procedure is equivalent to five cycles of home washing machine launderings.

Coating stability on treated fabrics were also tested by rinsing the sample with ethanol (purity: 99%) and ultrasonicating for 5 min at the same time, then drying at 60 °C for 50 min.

## 3. Results and Discussion

### 3.1. Plasma Operation Parameters

The optimum parameters for this plasma system were chosen by analyzing various processing parameters such as plasma cycle, precursor value, ionization gas flow rate, and so on.

#### 3.1.1. Plasma Cycle Time

**Figure 2:**
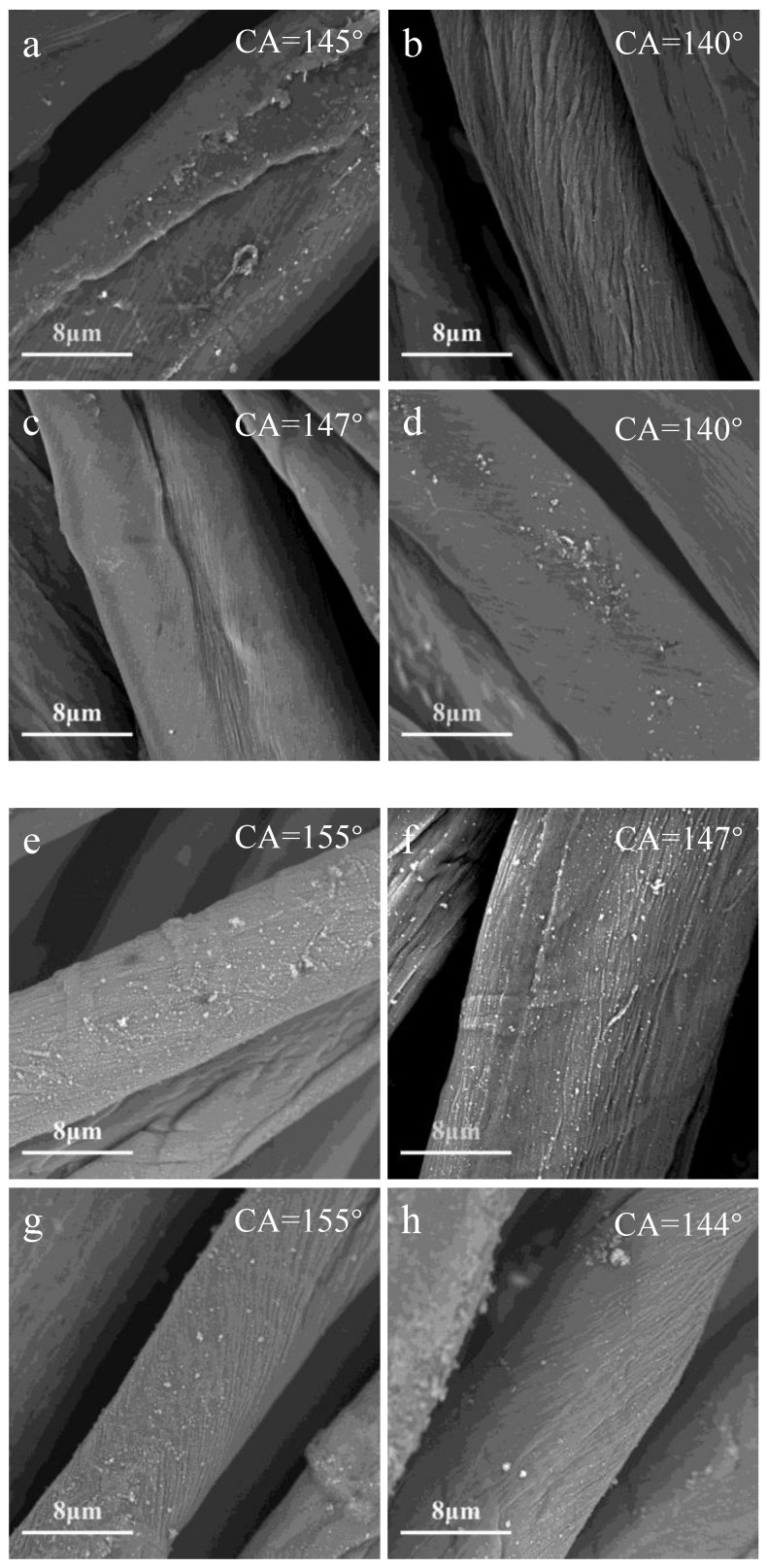
The effect of the plasma cycle time on the contact angle and surface morphology of N_2_ plasma-treated cotton fabric: (**a**) 20%; (**b**) 40%; (**c**) 60%; (**d**) 80%; O_2_ plasma-treated cotton: (e) 20%; (**f**) 40%; (**g**) 60%; (**h**) 80%.

The effect of the plasma cycle time on the surface morphology of plasma-treated cotton fabric is shown in Figure 2. In this experiment, the ionization gas (N_2_ and O_2_) flow rate was 2000 L/h, and the precursor injection rate value was kept at 15 g/h, In Figure 2a–h, the graphs showed that the surface deposition formation on the cotton fiber is different with different cycle times, changing the cycle time affects the energy of the ions, radicals, and electrons in the plasma, which can affect the formation and deposition processes. On the other hand, the interfacial properties of the coating do not change much, which the CA changed slightly with the different cycle time (see the pictures of the water drops on the cotton fabric and CA measurements in Figure S1, supplementary materials). It is also shown that the deposition is easier to form in the O_2_ plasma than in the N_2_ plasma, which results in higher contact angle in the O_2_ plasma treated substrate than in the N_2_ plasma treated substrate, even at the same precursor injection rate value. The SEM images show that the cotton fabric treated by 60% cycle time had a rougher surface than others cycle time, and particles were formed on a nanometer scale, which might have made the CA higher.

#### 3.1.2. Precursor Injection Rate Value

**Figure 3:**
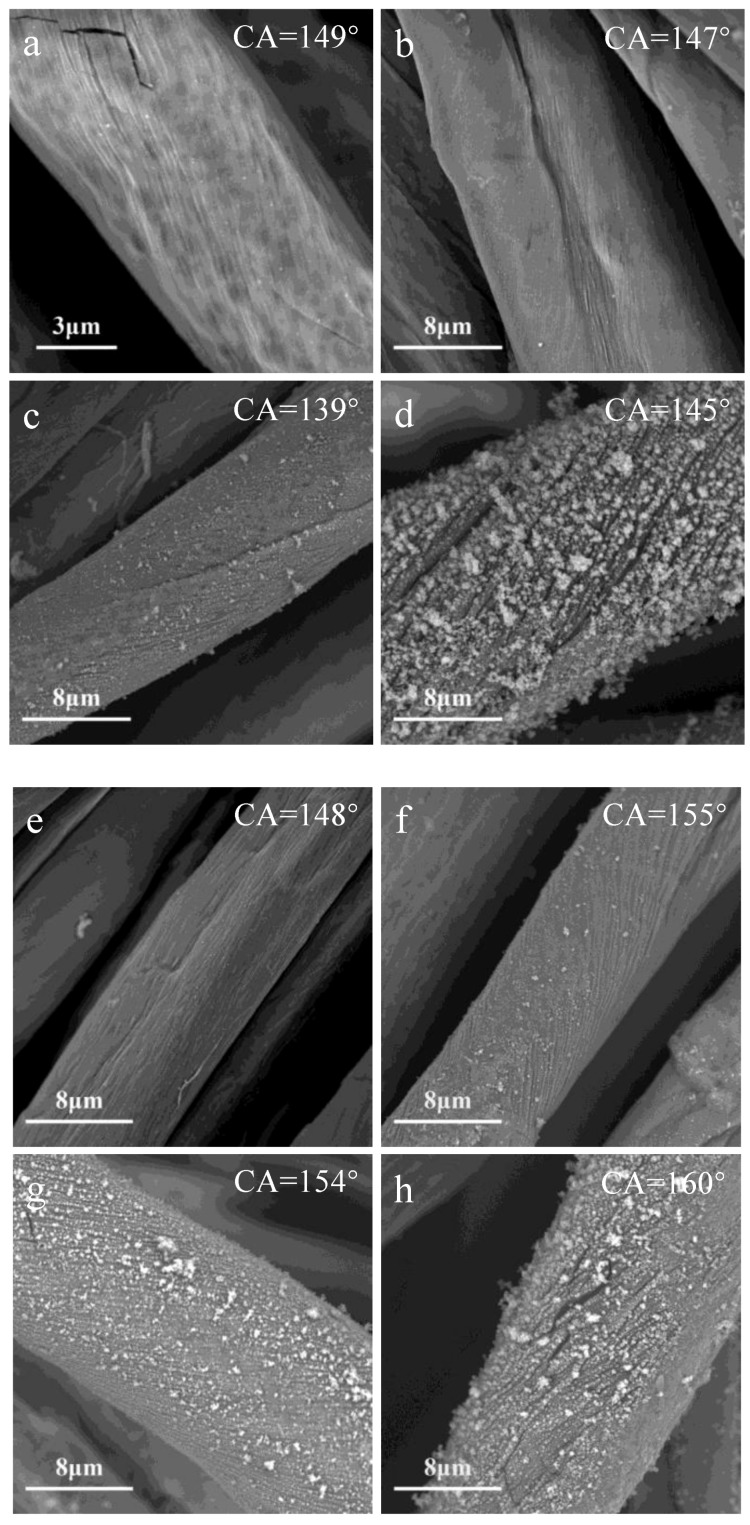
Scanning electron microscope (SEM) images and contact angle (CA) values of cotton fabric treated by different precursor value: N_2_ plasma (**a**) 10 g/h, (**b**) 15 g/h, (**c**) 20 g/h, (**d**) 30 g/h; O_2_ plasma (**e**) 10 g/h, (**f**) 15 g/h, (**g**) 20 g/h, (**h**) 30 g/h.

SEM images and CA values of cotton fabric treated by N_2_ plasma and O_2_ plasma with different precursor injection rate value are shown in Figure 3. In this experiment, the ionization gas flow rate was 2000 L/h. Previous literature have shown that the precursor injection rate affects particulate formation and deposition processes [21]. The formation of nanoparticles was attributed to the rapid monomer replenishment in the plasma discharge [22], as can be seen in Figure 3a–g, more and more nanoparticles are agglomerated with the increase of precursor injection rate value forming a bigger and denser coating. In our current study, the HMDSO deposition were formed in a bigger and denser layer with the increase of the precursor injection rate value. Figure 3 also showed that the precursor injection rate value influenced the contact angle only slightly, as the surface chemical composition were becoming very similar after the plasma treatment, with differences in surface roughness only. Figure 3 also depicted that the cotton fabrics treated by O_2_ plasma had a higher CA than the ones treated by N_2_ plasma (see the CA photographys and measurements in Figure S2, supplementary materials). We don’t have good understanding of this consistent difference yet, with only speculation that O_2_ plasma might have created a surface morphology more conducive to physical hydrophobicity in the coated layers (also see discussions in Section 3.2).

The wet-out time of the treated fabrics could not be measured, because the treated cottons were not wetted by water drops until those water drops completely evaporated.

**Figure 4:**
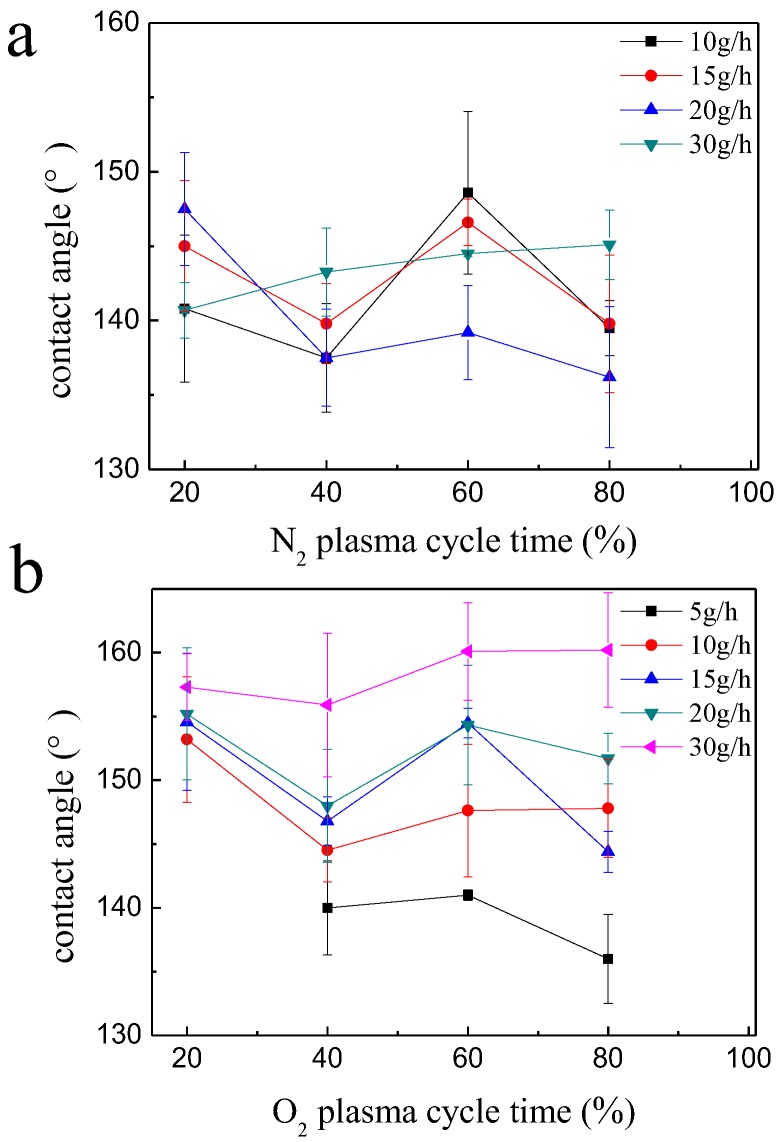
The CA of N_2_ plasma-treated cotton (**a**) and O_2_ plasma-treated cotton (**b**).

Figure 4 shows the combined effects of both plasma cycle time and precursor injection rate values on the CA of the treated cotton fabric. Figure 4a showed the effect of cycle time on the CA of cotton fabric coated in N_2_ plasma (see the pictures of the water drops and CA measurements in Figure S3a, supplementary materials), when the precursor injection rate value was 5 g/h, a complete layer of coating could not be formed on the surface of the fiber, hence the treated fabric did not show enough hydrophobicity. The same phenomenon also showed in the Figure 4b, which the treated fabric did not show the hydrophobicity when the precursor injection rate value and cycle time were 5 g/h and 20% (see the pictures of the water drops and CA measurements in Figure S3b, supplementary materials). At higher precursor injection rate and cycle time, the fabric surface became completely covered, and the fabrics showed superhydrophobicity. Hence, system was run at 60% plasma cycle time and 15 g/h precursor injection rate values in all following experiments.

#### 3.1.3. Ionization Gas Flow Rate

**Figure 5:**
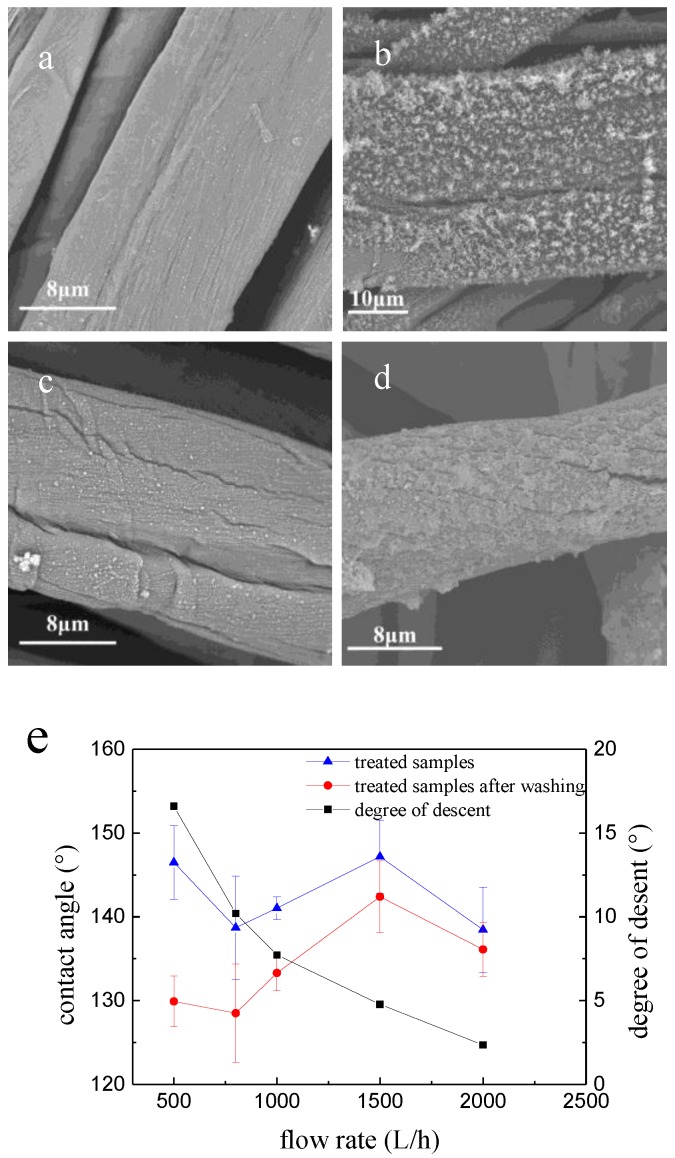
The effect of ionization flow rate on SEM images and CA values of N_2_ plasma-treated plain cotton fabric.

SEM images of N_2_ plasma-treated cotton fabric with different ionization gas flow rate of 2000 and 800 L/h are shown in Figure 5a,b. Coating stability on treated fabrics were also tested by rinsing the sample with ethanol (purity: 99%) and ultrasonicating for 5 min at the same time. The images of the samples after washing are shown in Figure 5c,d. As can be seen in the Figure 5b,d, the ionization gas flow rate has great effect on the surface morphology, the coating was homogeneous with nano-particles on the fiber surface at flow rate of 2000 L/h, and just appeared a few cracks having a slightly effect on the CA after washing, nevertheless, the coating was inhomogeneous at a lower 800 L/h flow rate, and was easy to wash out, making the treated fabric lower on CA measurements. Coating stability on treated fabrics were shown in Figure 5e that the lower of the N_2_ flow rate, the higher on the decreased CA readings after washing the N_2_ plasma-treated cotton fabric samples (see the pictures of the water drops on the cotton fabric and CA readings in Figure S4, supplementary materials). The lower N_2_ flow rate might have impacted the concentrations of active species radicals, electrons, ions in the plasma, as well as their life span and contact time with the substrate surface and the precursor molecules, with negative effect on surface deposition and polymerization on the fiber surfaces. In the following experiments, the plasma cycle time, precursor value and ionization gas flow rate were chosen at optimum parameters at 60%, 15 g/h and 1500 L/h, respectively.

As a reference note, the plasma discharge was seen to be unstable at 500 L/h gas media flow rate, so we chose to operate at higher flow rate to ensure the stable discharge of the plasma environment.

### 3.2. Surface Morphology Analysis

**Figure 6:**
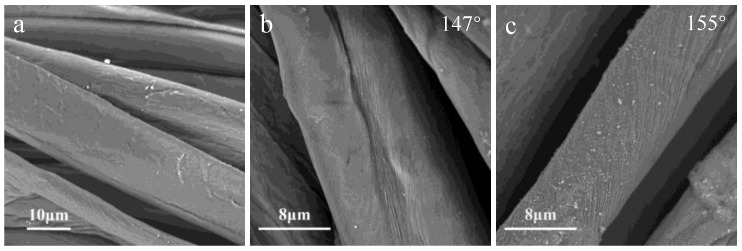
SEM images of samples: (**a**) untreated cotton; (**b**) N_2_ plasma-treated cotton; (**c**) O_2_ plasma-treated cotton.

Scanning electron microscopy (SEM) images of untreated and treated cotton fabrics are shown in Figure 6. The untreated cotton shows typical smooth surface with natural grooves, in contrast, both N_2_ plasma-treated and O_2_ plasma-treated cottons show homogeneous nano-particles deposited on the fiber surface. Surface morphology indicated that the hydrophobic coating was deposited onto the fiber surface with homogeneous nano-particles, making the treated cotton superhydrophobic. It is also obviously that the sizes of the nano-particle deposited on the O_2_ plasma-treated cotton are bigger than those of the N_2_ plasma-treated cotton fibers, which may be related to the higher contact angle measured (155°) than that of the N_2_ plasma-treated cotton fabrics. This rougher surface with nano-particles might have been responsible in creating higher levels of physical hydrophobicity than just the surface chemical compositions.

### 3.3. Chemical Composition and Structure Analysis

**Figure 7:**
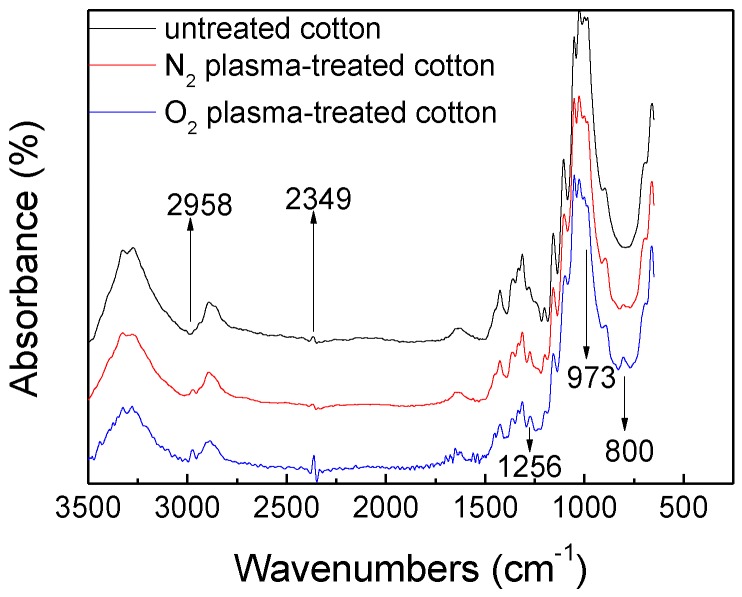
FTIR spectra of untreated cotton and plasma-treated cotton with N_2_ and O_2_ ionization gas.

The ATR-FTIR spectra of untreated cotton and plasma-treated cotton with N_2_ and O_2_ ionization gas are shown in the Figure 7. Absorbance intensities at 1030,1055, 1107 and 1316 cm^−1^ are assigned to a C–O stretch, asymmetric in-plane ring stretch, asymmetric bridge C–O–C and CH wagging vibrations [10], respectively, which is a typical infrared spectrum of untreated cellulose in cotton fabric. In Figure 6, characteristic peaks at 800 and 973 cm^−1^ are assigned to Si–O–Si bending vibration and Si–O–C symmetric stretching vibrations [14]. The peaks at 2958 and 1256 cm^−1^ assigned to CH_3_ asymmetric stretching and Si–CH_3_ symmetric bending are also observed in both N_2_ and O_2_ plasma treated samples, which are absent in the untreated cotton, indicated that Si–CH_3_ groups in the structure of the deposited coating formed by HMDSO plasma polymerization. FTIR spectra analysis indicates that deposited coating treated by plasma polymerization contains Si–O–Si, Si–O–C, CH_3_, Si–H_3_ groups, absent in the untreated cotton, all new radicals participate in the plasma polymerization reaction forming a layer of superhydrophobic coating.

### 3.4. EDS Analysis

**Figure 8:**
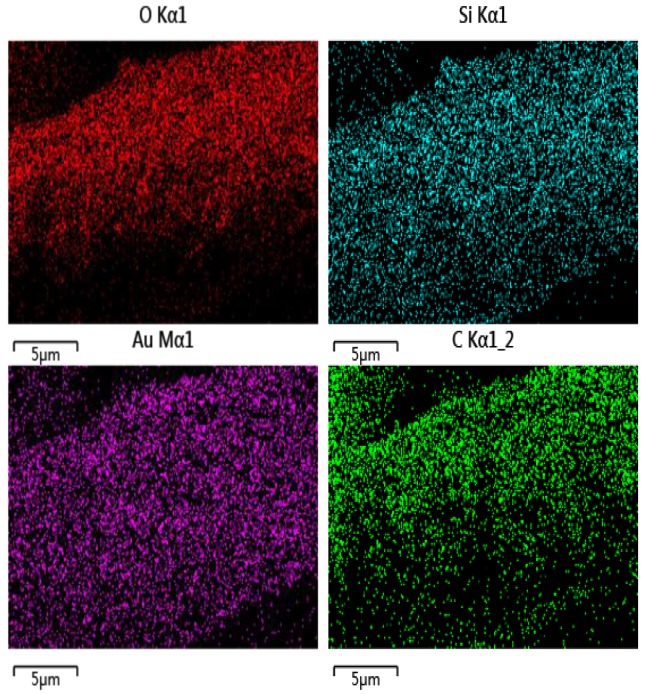

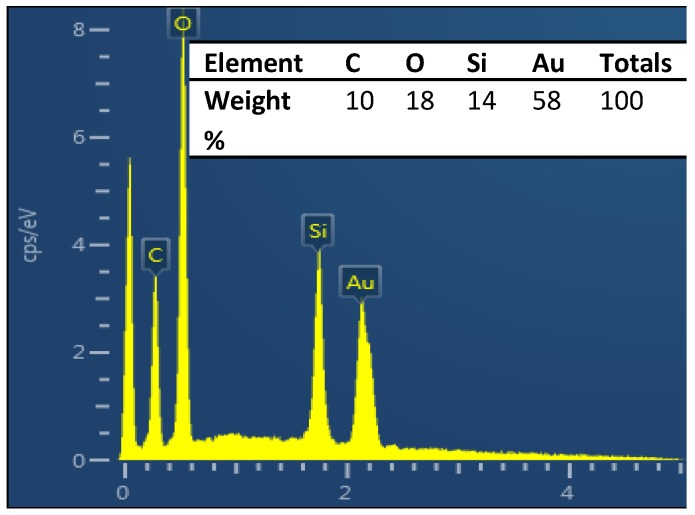
EDS mapping dots and element weight of the N_2_ plasma treated sample.

FE-SEM image and EDS mapping dots analysis of the Si elements, C, O, and Au obtained from protrusion pattern fiber surface coated by N_2_ plasma are shown in Figure 8. As shown in Figure 8, the mapping dots of Si elements, C, O, and Au tend to have a homogeneous distribution throughout the surface where it was deposited onto the superhydrophobic layer. It also shows that the proportion of silicon element among the four elements is 12%, which proves that silicon is introduced into the polymeric film coated on the fiber surface.

### 3.5. Durability Analysis

**Figure 9:**
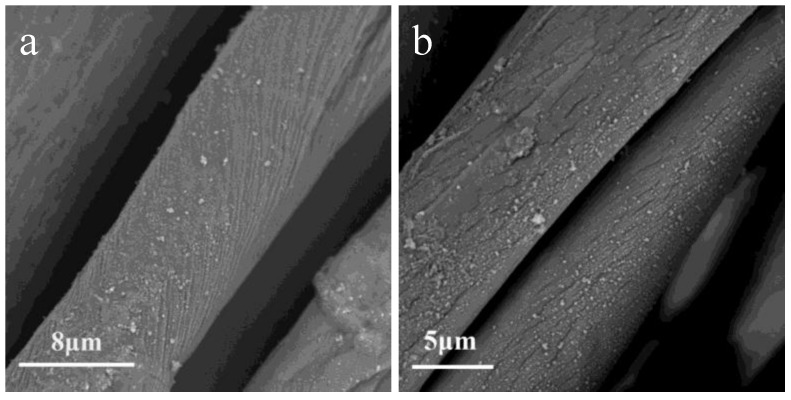
SEM images of O_2_ plasma treated sample (**a**), and treated samples after standard washing (**b**).

The deposited layers’ washing fastness of the O_2_ plasma-treated fabrics were given in Figure 9a,b, and the durability was evaluated by a standard washing procedure (AATCC 61 No. 1A). The CA of the treated fabric decreased by 3°–20°, while still all over 140°, indicating good durability in their hydrophobicity. Figure 9b showed some small pieces of coating were removed after washing, reducing the CA of the washed fabric slightly, but still maintaining enough coverage on the fiber surface to keep their hydrophobicity.

**Figure 10:**
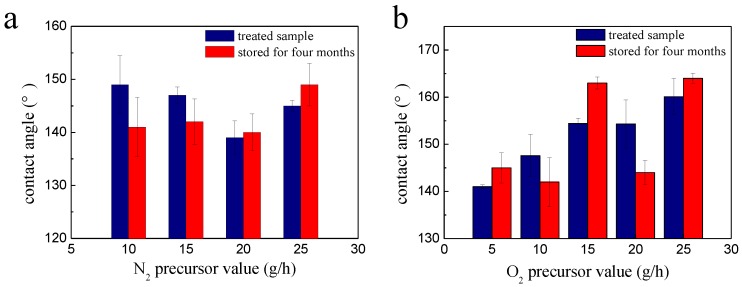
The durability of N_2_ plasma-treated cotton fabric (**a**) and O_2_ plasma-treated cotton fabric (**b**) after stored for four months.

Another important consideration in surface modification of organic matters is their aging stability, as it is known that surface dynamics in chemical chain movements over time may often lead to aged surfaces losing their hydrophobic characteristics. This phenomenon has been especially worrisome for plasma treated surfaces, as chemical compositions on the surface of polymers often fold and move into the bulk upon continued exposure to atmosphere environment. In Figure 10a,b, the contact angles of the cotton fabric treated by both of the N_2_ plasma and O_2_ plasma changed ±2°~10° after being stored for four months, which showed a prominent hydrophobicity character.

**Figure 11:**
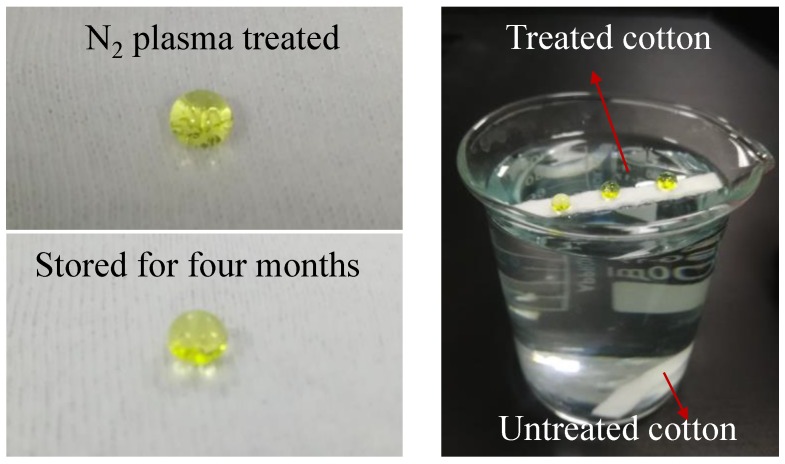
The pictures of a drop of colored water on the treated samples.

Figure 11a shows the colored water droplet on the treated cotton fabric surface, its spherical shape, and that it can still maintain this shape even though samples are stored in standard conditions for four months. The contact angle of the treated fabric stored for four months did change slightly, which was almost 160°, indicating a typical and stable superhydrophobicity character. Figure 11b indicated that the treated cotton fabric showed great hydrophobicity on both sides and can keep floating for a few days, while the untreated cotton immersed in water immediately, which also demonstrated that hydrophobic coating was polymerized on the opposite surface of cotton fabrics [23].

### 3.6. Air Permeability Analysis

**Figure 12:**
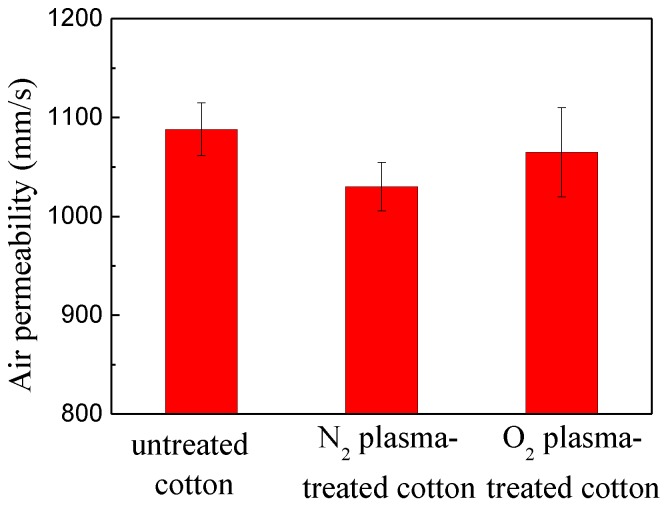
The air permeability of untreated, N_2_ plasma-treated cotton fabric and O_2_ plasma-treated cotton fabric.

The air permeability of untreated, N_2_ plasma-treated cotton fabric and O_2_ plasma-treated cotton fabric are shown in the Figure 12. The value of untreated cotton fabric was 1100 mm/s, and N_2_ plasma-treated cotton fabric and O_2_ plasma-treated cotton fabric were 1035 and 1065 mm/s, respectively. It can be stated that siloxane deposition in the form of nano-particles on cellulose fibers through N_2_ or O_2_ plasma treatment have little influence on the air permeability of the original cotton fabric.

## 4. Conclusions

In this paper, we have established that siloxane (HMDSO) grafting on cellulose fibers through atmospheric pressure N_2_ and O_2_ plasma treatment process is an effective technique to fabricate superhydrophobic cotton fabric with a high durability, allowing for a wide range of practical uses of the treated fabrics. The contact angle of the treated cotton fabric thus could reach more than 160°, with almost no change after storage in ambient conditions for four months, or even being subjected to rigorous laundering according to the AATCC standard methods. This surface treatment neither changes the fabric appearances nor the structural properties such as color or air permeability. We further explored the process parameters such as the siloxane precursor injection rate, ionization gas flow rate, and plasma cycle time to demonstrate an optimum process conditions for the graft-deposition layer formation on the surface of the cotton fabric. The surface modifications were further characterized in their nano-particle morphology by the SEM images, their organosilicone thin films composition having a homogeneous, highly crosslinked network within coated layers by EDS and ATR-FTIR analyses.

## Figures and Tables

**Table 1:** Operational parameters studied used in the experiment.

Operational Parameters	Unit	Range
Precursor value	g/h	5–25
Ionization gas flow rate	L/h	500–2000
Plasma cycle time	%	20–80

## Supplementary Materials

The following are available online at http://www.mdpi.com/2073-4360/10/4/460/s1.
