# Supplementary material for: Fabrication of Durably Superhydrophobic Cotton Fabrics by Atmospheric Pressure Plasma Treatment with a Siloxane Precursor
Source: Polymers (Basel). 2018 Apr 22;10(4):460. doi: 10.3390/polym10040460 (PMC6415397; doi:10.3390/polym10040460)
Supplement: Supplementary file 1 [file polymers-10-00460-s001.pdf]

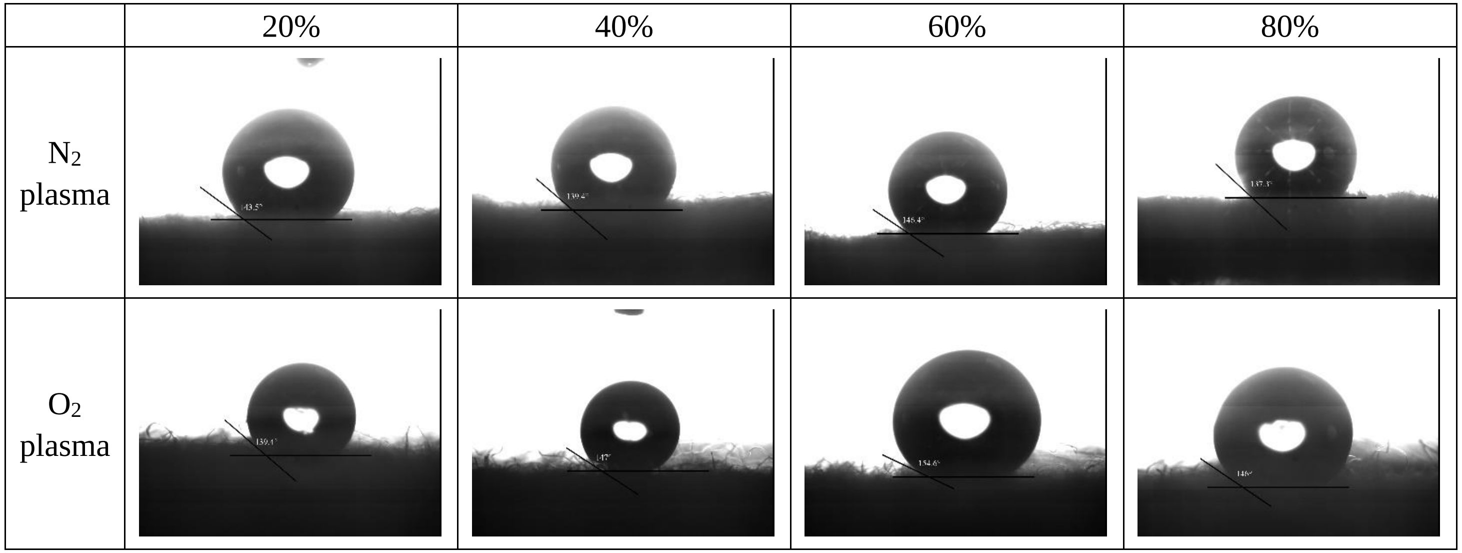

Fig. S1 The CA photography of N_2 and O_2 plasma-treated cotton fabric: (a)20%; (b) 40%; (c) 60%; (d) 80%;

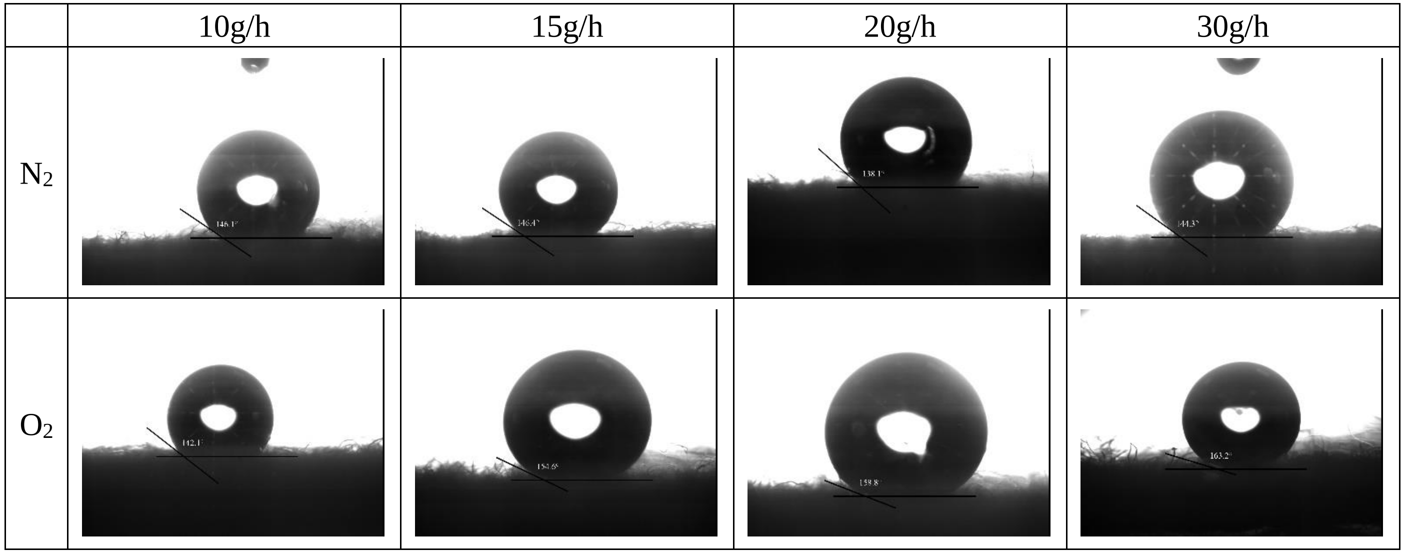

Fig. S2 CA values photograph of cotton fabric treated by different precursor value: N_2 plasma and O_2 plasma (a) 10g/h, (b) 15g/h, (c) 20g/h, (d) 30g/h

	20%	40%	60%	80%
10g/h	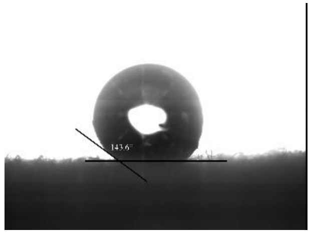	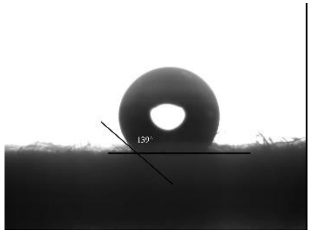	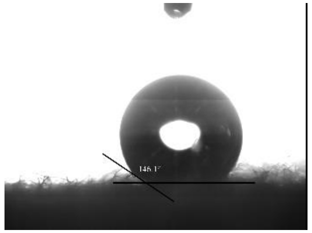	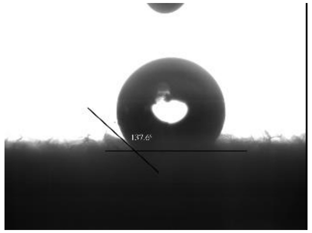
15g/h	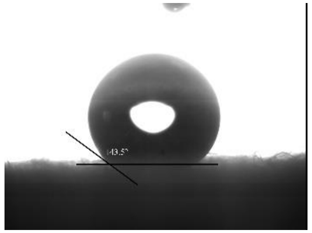	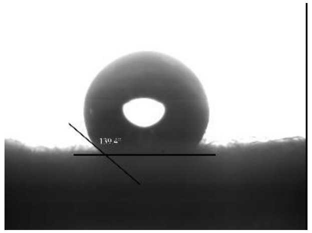	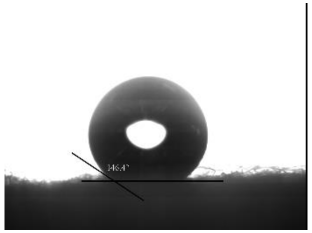	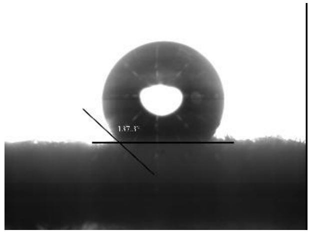
20g/h	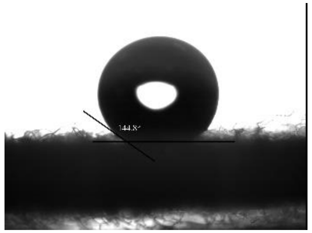	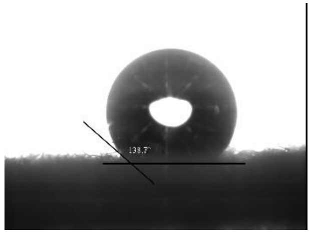	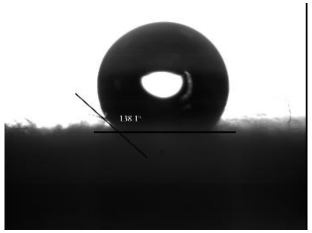	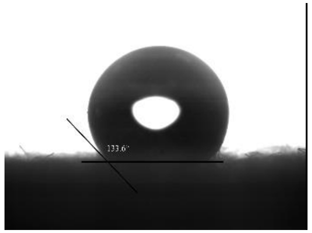
30g/h	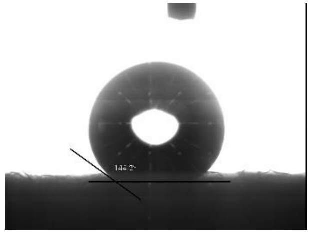	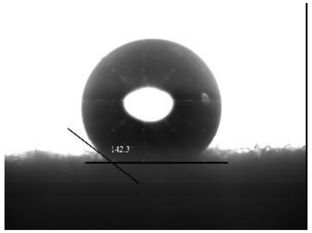	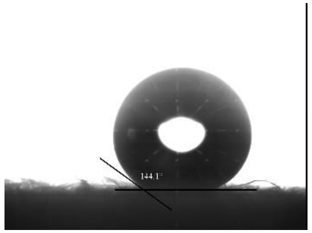	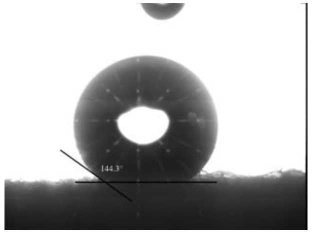

a

	20%	40%	60%	80%
5g/h		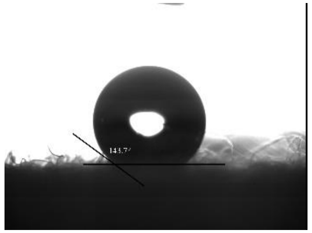	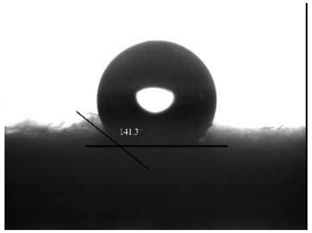	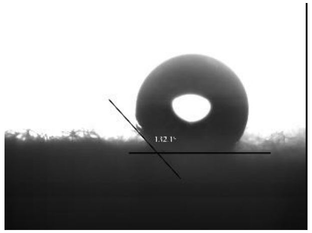
10g/h	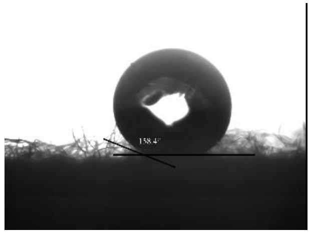	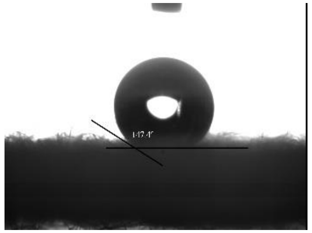	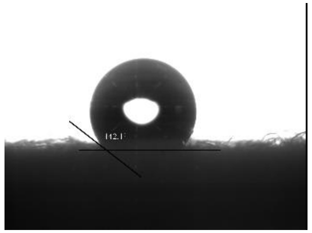	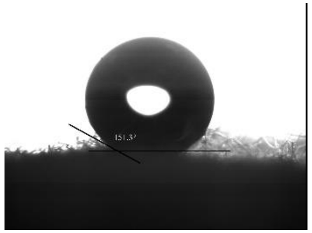
15g/h	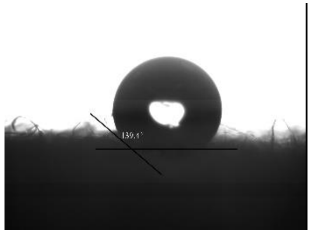	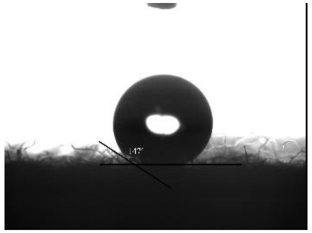	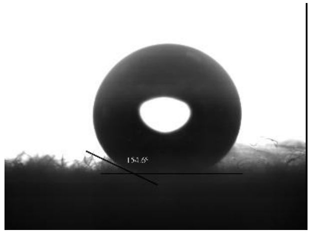	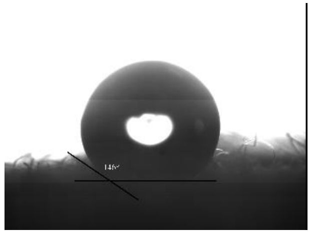
20g/h	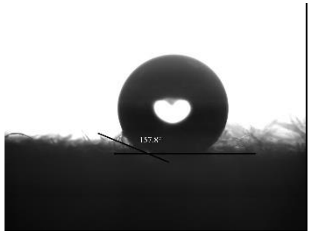	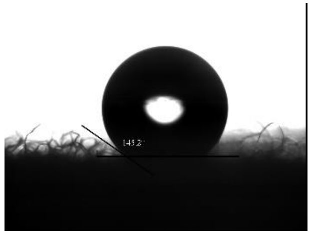	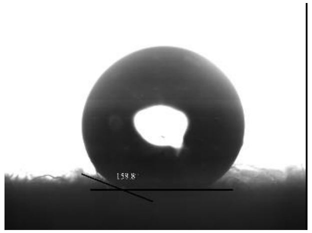	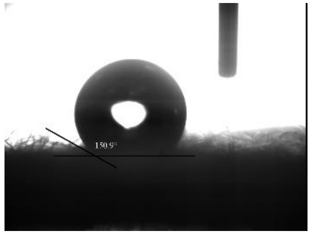

30g/h	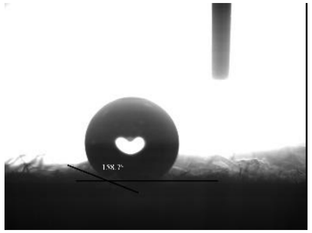	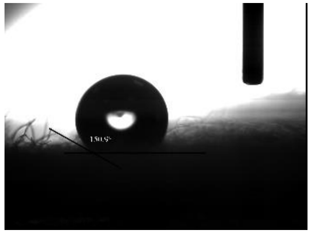	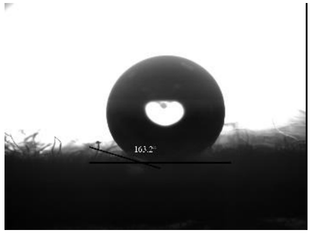	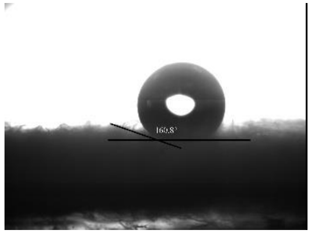

b

Fig. S3 The CA photograph of N_2 plasma-treated cotton (a) and O_2 plasma-treated cotton (b)

	500	800	1000	1500	2000
Treated samples	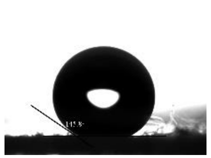	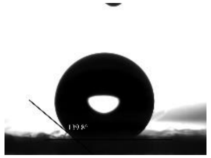	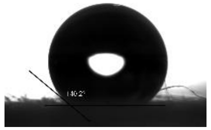	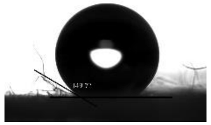	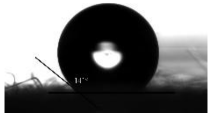
Treated samples after washing	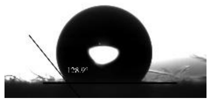	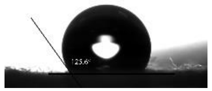	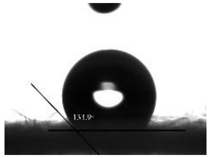	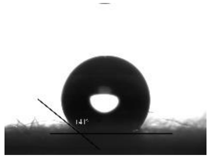	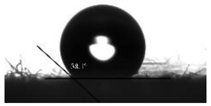

Fig. S4 The CA values photograph N_2 plasma-treated plain cotton fabric
